# Comparison between cryoballoon double stop and single stop in patients with paroxysmal atrial fibrillation

**DOI:** 10.1016/j.ipej.2023.06.005

**Published:** 2023-07-05

**Authors:** Takashi Yamada, Masato Murakami, Nobuhisa Kodera, Takahiro Hayashi, Takafumi Koyama, Shingo Mizuno, Shigeru Saito

**Affiliations:** Department of Cardiology, Shonan Kamakura General Hospital, Kamakura, Japan

**Keywords:** Cryoballoon ablation, Atrial fibrillation, Double stop, Catheter ablation

## Abstract

**Background:**

Currently, cryoballoon (CB) thawing after single stop is generally performed. Previous research had reported that long thawing time using a single stop affects pulmonary veins tissue injury. However, it is uncertain whether CB thawing after single stop affects clinical outcomes.

**Objective:**

This study aimed to clarify clinical significance of CB thawing in patients with paroxysmal atrial fibrillation.

**Methods:**

Two hundred ten patients with paroxysmal atrial fibrillation who underwent CB from January 2018 to October 2019 were analyzed. We compared the clinical outcomes of patients whose CB applications were completely stopped with only the double stop technique (DS group, n = 99) and patients with single stop (SS group, n = 111). In DS group, we performed double stop technique for all CB application regardless of phrenic nerve injury or the temperature of esophagus.

**Results:**

The atrial arrhythmia free-survival rate at 2 years after CB was significantly lower for the DS group than the SS group (76.8% vs 87.4%; p = 0.045). Complications occurred in 2 patients from the DS group and no complications were observed in patients from the SS group (p = 0.13). Mean procedural time was shorter in the DS group than in the SS group (53.1 vs 58.1 min; p = 0.046)

**Conclusion:**

DS group had higher recurrence rate than SS group. There was no significant difference regarding safety between both the groups. We found that the thawing process after single stop is very important for CB application.

## Teaser

In this study, we compared patients whose CB applications were completely stopped with only the double stop technique (DS group, n = 99) and those whose CB applications were stopped with single stop (SS group, n = 111).

## 1 Introduction

Recently, cryoballoon (CB) ablation for atrial fibrillation (AF) has been performed as a valid alternative to radiofrequency ablation, and a comparable efficacy and safety were demonstrated in a previous prospective randomized study [1].

CB standard applications require that the balloon temperature reach 20 °C prior to deflation.

In general, this balloon deflation system is called single stop (SS). During CB thawing time, extracellular ice melt causes an osmotic shift, the water then flows into the cells, resulting in tissue injuries due to fluid accumulation. Growth of intracellular ice crystals continues to exacerbate cellular damage when thawing time is less than −20 °C [2].

On the other hand, CB ablation is associated with a risk of phrenic nerve palsy (PNP) and esophageal thermal decrease. When we encounter PNP and esophageal thermal decrease during pulmonary vein isolation (PVI) with CB, we sometimes need to stop balloon deflation immediately [3,4].

This immediate balloon deflate system is called double stop (DS). DS technique enforces balloon deflation at the end of the ablation and accelerate tissue rewarming. Currently, we only use DS technique for emergent prevention of PNP and esophageal thermal decrease. Aryana et al. reported that thaw time at 0 °C of more than 10 s significantly predicts PVI durability [5]. They suggested that the duration of thawing time is important in SS. In previous studies, Ghosh et al. had reported that immediate balloon deflation results in more rapid tissue warming, causes no adverse events. Moreover, simulations suggest that immediate balloon deflation is unlikely to damage the endocardium [6]. Andrade et al. also reported that no differences were observed in the proportion of circumferential transmural ablation lesions, mean lesion depths, and mean and maximal neointimal thickness with passive (SS) versus active (DS) deflation techniques in dogs [2]. Combined, these results imply that forcible balloon deflation (DS) has no associated safety problems. However, studies comparing SS and DS directly are elusive, hence, the clinical validity is indemonstrable. Therefore, whether thawing process is necessary for CB application is clinically uncertain. We hypothesized that double stop for all CB application regardless of phrenic nerve injury or temperature of esophagus is effective, safe and comparable to single stop, moreover procedure time may shorten because of rapid balloon deflation.

The present study aimed to clarify the clinical outcome and safety of the CB double stop technique in patients with paroxysmal atrial fibrillation (PAF).

## 2 Methods

### 2.1 Study design

**Fig. 1:**
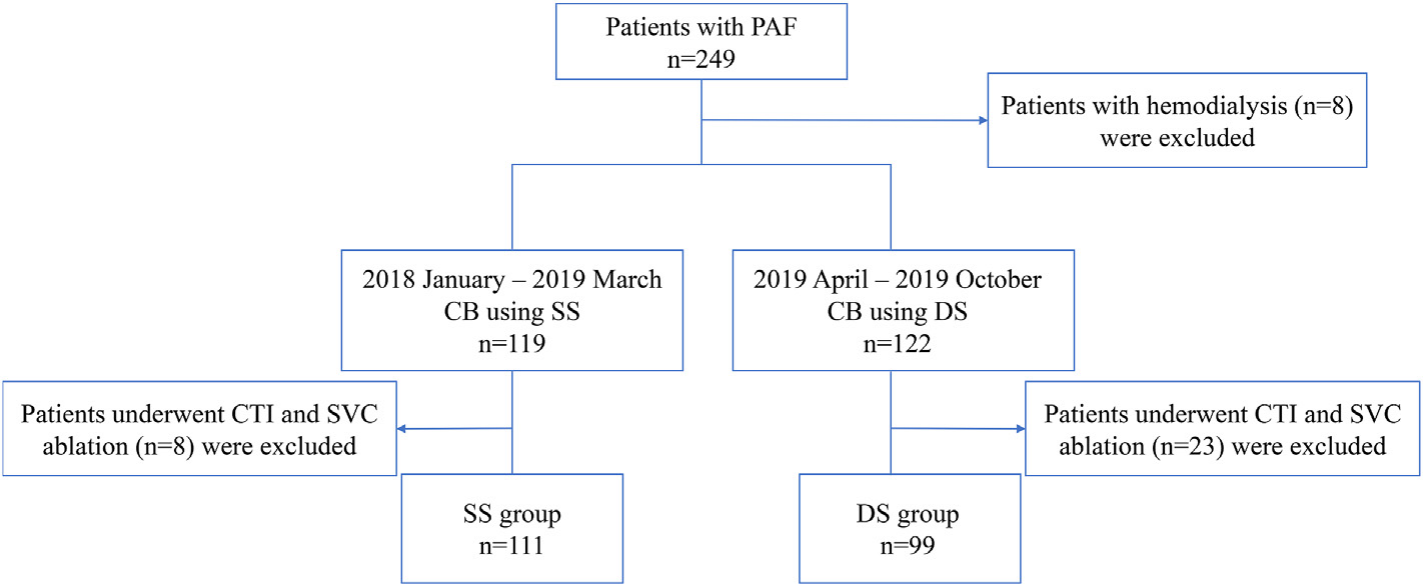
Flowchart of the study procedure.

Two hundred forty-nine patients with PAF who underwent first PVI with CB from January 2018 to August 2019 in Shonan Kamakura General Hospital undergoing CB ablation were selected. Eight patients with hemodialysis and 31 patients who underwent additional procedures such as cavo-tricuspid isthmus (CTI) linear ablation and superior vena cava (SVC) isolation were excluded. Two hundred ten patients were then analyzed retrospectively (Fig. 1).

We performed standard procedure for using single stop from January 2018 to March 2019. From April 2019, we started to use the double stop technique for all CB application because in an expectation of reducing procedure time.

In all patients, PVI was performed exclusively with a 28-mm CB (Arctic Front Advance, Medtronic) and 3-min freeze strategy.

All patients gave their written informed consent. The data, analytic methods, and study materials will not be made available to other researchers for purposes of reproducing the results or replicating the procedure as the original data includes the patient's personal information.

The primary outcome was defined as freedom from a documented recurrence of atrial tachyarrhythmia (ATA) lasting >30 s excluding 3-month blanking period. This primary outcome was analyzed 2 years after the first CB ablation. Freedom from a composite outcome of ATA recurrence or a prescription of an AAD (class Ⅰ or Ⅲ) was also assessed.

Safety outcome was defined as complications related to the procedure, such as pericardial tamponade, PNP, cerebrovascular events, and groin site complications.

Procedure time was calculated from the first puncture to completion of sheath removal and the thawing time was calculated from the end of application until CB temperature reached >20 °C.

### 2.2 Ablation protocol

Preprocedural cardiac enhanced computed tomography was performed to evaluate the cardiac anatomy. The procedure was performed under deep sedation with propofol and pentazine. A nasogastric thermometer (Esophastar, JAPAN LIFELINE, Japan) was inserted to measure the esophageal temperature during CB ablation. 3000 IU of heparin was administered following the venous access.

An activated clotting time of 300–350 s was maintained with a borus infusion of heparin during procedure. A single transseptal puncture was performed using a radiofrequency needle and 8-Fr long sheath (SL-0, SJM, Minneapolis, Minnesota) under the fluoroscopic guidance. After the Brockenbrough procedure, we injected additional 3000 IU of heparin. The transseptal sheath was exchanged over a guidewire for a 15-Fr steerable sheath (Flexcath Advance, Medtronic, Minnesota). A spiral mapping catheter (Achieve, Medtronic) was used to advance the 28-mm CB into the PV for support and to map the PV potentials. Following the verification of complete sealing with a contrast medium injection, freezing was applied [7]. The freezing cycle was defined as the time to isolate plus 120 s, with a maximum of 240 s. If PVI could not be achieved within the sufficient freezing timeframe of >120 s, additional second or more application were performed. During the CB application, the continuous motor action potential (CMAP) was monitored. In cases with either a minimum temperature under −55 °C of left PVs and −60 °C of right PVs, an esophageal temperature under 18 °C [8], or weakening or loss of diaphragmatic movement, double stop technique was performed regardless of SS group [9]. If PV potential did not disappear in spite of several applications, we performed touch up ablation with a 4 mm-tip irrigated ablation catheter (FlexAbility, Abbott, Chicago, IL).

After PVI, 0.1 μg/kg of isoproterenol was intravenously injected. If any atrial tachycardia was found, we performed additional ablation. When non-PVI foci were located in the superior vena cava (SVC), the SVC was electrically isolated. In case that preoperative or perioperative atrial flutter was found, Cavo-tricuspid isthmus (CTI) ablation was performed.

Dormant PV conduction was intended to be induced with adenosine triphosphate (20 mg) under isoprotelenol infusion. If dormant PV conduction was captured, an additional CB application or touch up ablation was performed. We did not have a resting time after these procedures.

### 2.3 Follow-up

Anticoagulation was discontinued three months after the index procedure. All patients were followed on an outpatient basis at one, three, six, 12 months and 24 months after the ablation procedure. Moreover 12-lead electrocardiography (ECG) was performed at every visit and Holter ECG monitoring was performed at three months and 12 months after ablation. We also consulted their family care doctors about arrhythmia recurrence. If patients had a symptom even if no AF was documented in the Holter ECG, portable ECG monitoring was used.

### 2.4 Statistical analysis

Data are expressed as means ± standard deviations for continuous variables and as frequencies and percentages for categorical variables. To compare the two groups, chi-squared analysis or Fisher's exact test was used for categorical variables and an unpaired *t*-test or Wilcoxon analysis was used for continuous variables. The follow-up period was calculated from the date of the procedure to the date of primary endpoint or censoring.

The Kaplan-Meier method was used to analyze any unadjusted ATA recurrence rates, which were compared using the log-rank test. A two-side p value less than 0.05 was considered statistically significant. All statistical analyses were performed using SPSS 26.0 (IBM Inc., Armonk, NY, USA).

## 3 Results

### 3.1 Baseline characteristics

Among 210 patients who underwent CB for PAF, mean age was 70.5 ± 10.0 years in the DS group and 68.1 ± 11.7 years in SS group (p = 0.1). The proportion of men in DS group were 69.7% and SS group were 55.0% (p = 0.03). βblocker was used 23.2% in DS group and 11.7% in SS group before procedure (p = 0.03) (Table 1).

**Table 1:** Baseline characteristics.

	baseline characteristics		
	DS group(n = 99)	SS group(n = 111)	p value
Age, year	70.5(±10.0)	68.1(±11.7)	0.1
Male, n (%)	69(69.7)	61(55.0)	0.03
Height, cm	165.5(±9.0)	163.3(±9.2)	0.09
Weight, kg	64.5(±14.0)	61.7(±11.0)	0.11
BMI, kg/m2	23.3(±3.6)	23.0(±3.1)	0.48
Hypertension, n (%)	62(62.6)	68(61.3)	0.63
Dyslipidemia, n (%)	27(27.2)	35(31.5)	0.5
DM, n (%)	15(15.6)	11(9.9)	0.25
prior CHF, n (%)	4(4.0)	4(3.6)	0.87
βblocker, n (%)	23(23.2)	13(11.7)	0.03
AAD, n (%)	6(6.1)	6(5.4)	0.84
stroke, n (%)	3(3.3)	2(1.8)	0.56
CHADs2 score	1.3(±0.9)	1.1(±0.9)	0.19
LAD, mm	38.2(±6.2)	37.2(±5.2)	0.1
LVEF, %	63.6(±7.3)	64.6(±6.7)	0.21
LAV, ml	67.3(±20.9)	58.5(±18.4)	<0.01
LAVi, ml/m2	39.5(±11.7)	35.3(±11.6)	0.023
Cr, mg/dL	0.93(±0.21)	0.83(±0.17)	<0.01
eGFR	59.9(±11.4)	64.6(±12.9)	<0.01
BNP, pg/mL	103.4(±95.3)	65.2(±70.3)	0.06

BMI: body mass index, DM: diabetes mellitus, CHF: congestive heart failure, AAD: anti arrhythmic drag, LAD: left atrial diameter, LVEF: left ventricular ejection fraction, LAV: left atrial volume, LAVi: left atrial volume index, Cr: creatinine, eGFR: estimated glemerular filtration rate, BNP: brain natriuretic peptide.

### 3.2 Procedural characteristics

Acute success rate was 100% in both groups. The mean procedure time was 53.1 ± 18.6 min in DS group and 58.1 ± 17.3 min in SS group (p = 0.046). Thawing time in each PVs was as follows (LSPV: 27.9 ± 4.9 vs 61.7 ± 16.7 min; p < 0.001, LIPV: 25.4 ± 5.3 vs 46.3 ± 13.5 min; p < 0.001, RSPV: 28.5 ± 4.6 vs 58.8 ± 13.5 min; p < 0.001, RIPV: 26.7 ± 4.5 vs 49.0 ± 16.1 min; p < 0.001). Five patients (5.1%) in DS group and 8 patients (7.2%) received touch-up ablation (p = 0.52) (Table 2).

**Table 2:** Procedural characteristics.

	procedere characteristics		
	DS group(n = 99)	SS group(n = 111)	p value
procedure time, min	53.1(±18.6)	58.1(±17.3)	0.046
LSPV application, number	1.4(±0.91)	1.6(±1.2)	0.16
LSPV diameter, mm	18.8(±2.9)	19.0(±3.1)	0.97
LSPV time to isolation, sec	51.0(±25.6)	58.4(±28.5)	0.07
LSPV minimum temparature, °C	−47.9(±4.1)	−49.3(±4.2)	0.02
LSPV thawing time, sec	27.9(±4.9)	61.7(±16.7)	<0.01
LIPV application, number	1.3(±0.52)	1.3(±0.68)	0.67
LIPV diameter, mm	16.6(±2.8)	16.8(±2.7)	0.65
LIPV time to isolation, sec	34.0(±29.3)	25.8(±14.9)	0.09
LIPV minimum temparature, °C	−43.3(±4.3)	−42.2(±21.7)	0.63
LIPV thawing time, sec	25.4(±5.3)	46.3(±13.5)	<0.01
RSPV application, number	1.2(±0.56)	1.3(±0.53)	0.54
RSPV diameter, mm	21.0(±2.4)	19.9(±3.2)	0.12
RSPV time to isolation, sec	37.7(±20.8)	34.0(±18.9)	0.23
RSPV minimum temparature, °C	−51.7(±5.6)	−52.3(±5.2)	0.44
RSPV thawing time, sec	28.5(±4.6)	58.8(±13.5)	<0.01
RIPV application, number	1.4(±0.92)	1.6(±1.1)	0.2
RIPV diameter, mm	18.2(±2.8)	17.8(±2.9)	0.42
RIPV time to isolation, sec	50.9(±27.9)	54.3(±28.3)	0.51
RIPV minimum temparature, °C	−47.9(±6.2)	−48.7(±5.9)	0.35
RIPV thawing time, sec	26.7(±4.5)	49.0(±16.1)	<0.01
Touch up, n (%)	5(5.1)	8(7.2)	0.52

LSPV: left superior pulmonary vein, LIPV: left inferior pulmonary vein, RSPV: right superior pulmonary vein, RIPV: right inferior pulmonary vein.

### 3.3 Freedom from any atrial tachy arrhythmia

Twenty-nine patients (26.1%) in the SS group and 19 patients (19.2%) in the DS group dropped out and could not be followed-up. Seven patients (6.3%) in SS group and 10 patients (10.1%) in DS group received portable ECG monitoring (p = 0.31).

**Fig. 2:**
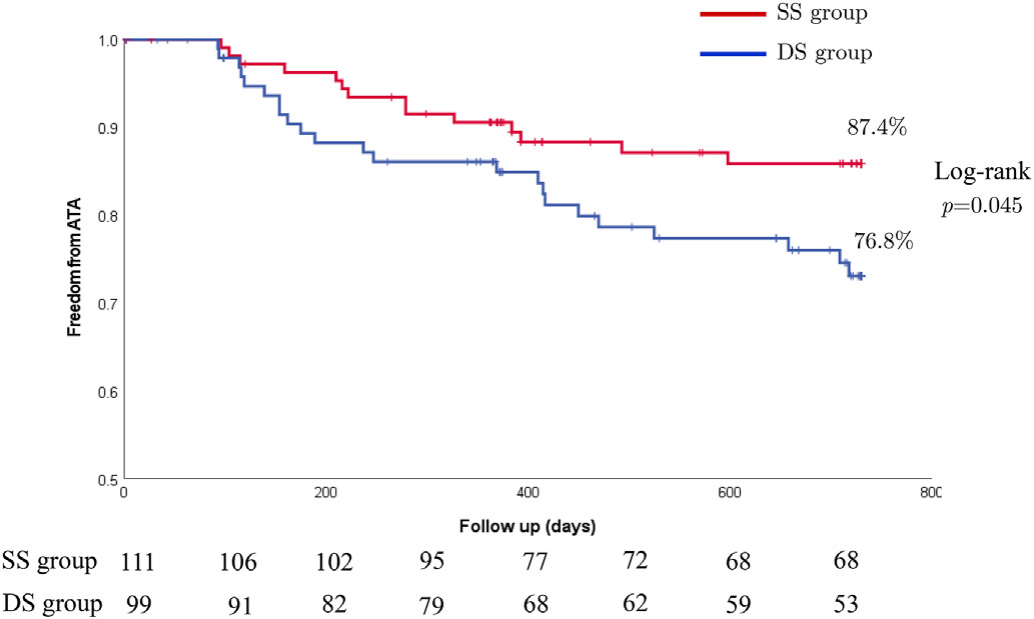
Freedom from a documented recurrence of any atrial tachyarrhythmia (ATA) on 2 years follow up.

**Fig. 3:**
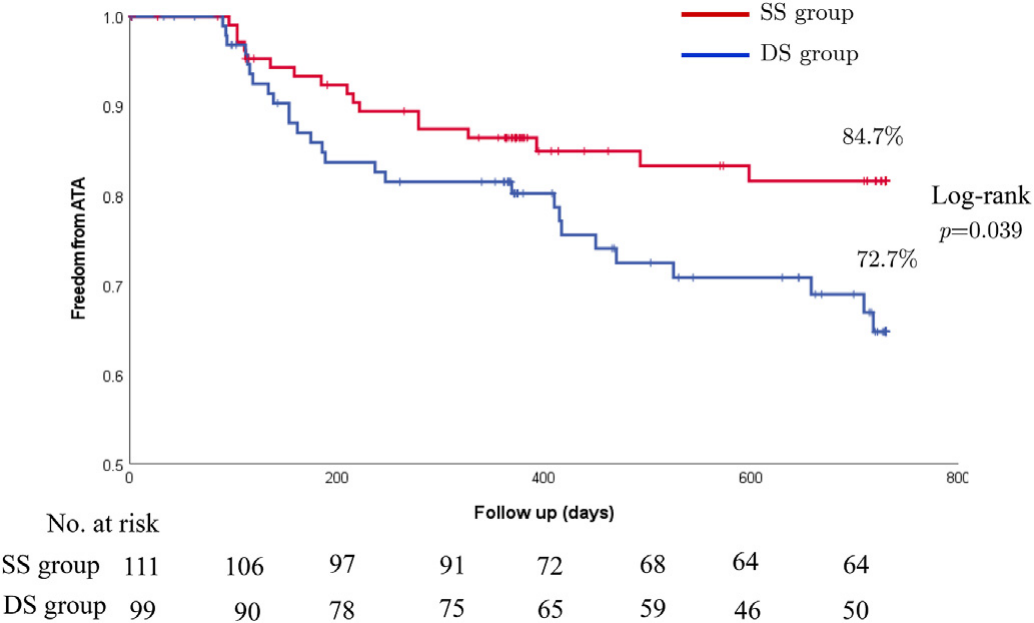
Freedom from a composite outcome of a documented recurrence of any atrial tachyarrhythmia (ATA) or a prescription of antiarrhythmic drugs on 2 years follow up.

The recurrence rate of ATA 12 month was 85.9% (14 out of 99 patients) in DS group and 85.9% (10 out of 111 patients) in SS group (p = 0.19). The recurrence rate of ATA 24 months after the first ablation session was 76.8% (23 out of 99 patients) in DS group and 87.4% (14 out of 111 patients) in SS group (p = 0.042) (Fig. 2). In addition, freedom from a composite outcome of ATA recurrence or a prescription of an AAD (class Ⅰ or Ⅲ) was 72.7% (27 out of 99 patients) in DS group and 84.7% (17 out of 111 patients) in SS group (p = 0.039) (Fig. 3).

### 3.4 Procedure related complications

Two patients in DS group and no patient in SS group had any procedure-related complications (Table 3). One patient had phrenic nerve palsy in the DS group. Groin site complications, which included pseudoaneurythm required endovascular treatment occurred in one patient in the DS group.

**Table 3:** Procedure-related complications.

	Complication		
	DS group	SS group	*p value*
Phrenic nerve palsy, n	1	0	0.29
Esophageal complication, n	0	0	
Cardiac tamponade, n	0	0	
Thromboembolism, n	0	0	
Groin site complication, n	1	0	0.29

## 4 Discussion

### 4.1 Main findings

This is the first report that performed the DS technique for all PVs and compared the clinical outcome between the DS and the SS group. In this study, a composite outcome of the recurrence rate of any ATA in DS group was significantly higher than the SS group. These findings suggest that thawing process after single stop is necessary for CB application. Thawing process included tissue injury due to fluid accumulation due to intracellular and extracellular osmotic pressure, and further tissue injury due to ice recrystallization as the temperature was less than −20 °C. Generally, frostbite is commonly treated using fast defrosting, and past studies have shown that quick thawing of cells has a preservative effect [10]. However, it is unknown whether thawing time of 30 s results in an improvement of frostbite in PVs. Thus, further studies are needed.

We should remember that performing DS means eliminating these important processes. Although we were not aware of thawing process clinically, it played an important role in lesion durability. As evidence to support importance of thawing, recurrence rate at 1year was not significantly different between DS group and SS group. However, arrhythmia recurrence started to diverge after 1 year. Finally, recurrence rate at 2 years follow up was significantly different between the DS group and SS group. These findings suggested that acute lesion of DS technique is not inferior to SS, however the long-term lesion durability is inferior. In addition, the acute PV success that required touch-up ablation during the index procedure had no significant difference between the two groups (7.2% in SS group vs 5.1% in DS group; p = 0.52). This result might show that acute lesion of DS technique is equivalent to SS.

Aryana et al. reported that time to isolation of less than 60 s and interval thaw time at 0 °C of more than 10 s significantly predict PVI durability [5]. Miyazaki et al. also reported that thawing time to 0 °C and 15 °C associated with durable PVI especially in right PVs [11]. Regarding these report, shorter time to isolation and longer thawing time is an important predictor of PVI durability. In our study, time to isolation in each PVs between the two groups was less than 60 s. Thus, we thought impact of time to isolation was small. When it comes to thawing time, previous studies have assessed only SS. These results highlight the need for more appropriate PV occlusion. The new findings of our study highlighted that even when the time to isolation is fast and PV occlusion is appropriate, forcible balloon deflation due to DS negatively affects the PVI lesion durability.

Bradley et al. and Giacomo et al. reported that freedom from AF and symptomatic atrial flutter/atrial tachycardia after CB ablation for paroxysmal atrial fibrillation was 70.8% and 63% at 24 months [12,13]. In Japan, a recent study showed that freedom from ATA was 86.7% at 24 months [14]. Our result after the first ablation session for PAF showed 76.8% in the DS group and 87.4% in the SS group, thus it was comparable to those reported by the previous study.

Procedure time in-line with our expectations. DS saved an average of 30 s per procedure. Considering the average of 1.5 applications per PV, all PVI saved about 3 min. Our results indicated that there was a difference of approximately 5 min in the procedure time between the two groups (53.1 vs 58.1 min; p = 0.046). However, a 5-min difference might not be much importance in clinical practice.

Finally, we excluded additional CTI ablation and SVC isolation from this study. Thus, our results could not be extended to those cases.

### 4.2 Complications

Procedure-related complications were not significantly different between the DS and SS group. We assumed that the DS technique, forced to deflate balloon from PV might result in some adverse events. For example, tissues inhered CB embolize due to forcibly deflation. However, such findings were not detected in our study.

Previous study also reported that phrenic nerve palsy occurred at 2.7% and groin site complications at 1.9% and stroke at 0.5% in CB group [1]. Complication rates in our study were not contradictory compared to past trials.

### 4.3 Limitations

This study has several limitations. First, this study was a single center, retrospective study. Sample size was small. Second, AF recurrence was underestimated. We followed at one, three, six, 12 months and 24 months after the ablation procedure. Moreover 12-lead electrocardiography (ECG) was performed at every visit and Holter ECG monitoring was performed routinely at three months and 12 months post ablation. However, we did not use any loop recorder, thus AF recurrence was detected at lower levels. Third, SS group were enrolled from January 2018 to March 2019, DS group were enrolled from April 2019 to August 2019. Therefore, we could not evaluate two groups simultaneously. Fourth, several patients dropped out between one year and two year of follow up. Thus, freedom from recurrence rate were lacking accuracy. Finally, the relationship between 2nd session and PV reconnection was unknown because of the small number of patients that underwent 2nd session. Further data were needed to determine if thawing time was involved in PV reconnection.

## 5 Conclusions

DS group had higher recurrence rate than SS group. We found that thawing process after single stop is very important for CB application.

## Funding sources

This research did not receive any specific grant from funding agencies in the public, commercial, or not-for-profit sectors.

## Declaration of competing interest

The authors declare no conflicts of interest associated with this manuscript.
